# Increased CX3CL1 in cerebrospinal fluid and ictal serum t-tau elevations in migraine: results from a cross-sectional exploratory case-control study

**DOI:** 10.1186/s10194-024-01757-8

**Published:** 2024-04-02

**Authors:** Marie Süße, Christine Kloetzer, Sebastian Strauß, Johanna Ruhnau, Lucas Hendrik Overeem, Merle Bendig, Juliane Schulze, Uwe Reuter, Antje Vogelgesang, Robert Fleischmann

**Affiliations:** 1 Department of Neurology, University Medicine Greifswald, Ferdinand-Sauerbruch-Str. 1, 17475 Greifswald, Germany; 2 Department of Neurology With Experimental Neurology, Charité - Universitätsmedizin Berlin, Charitéplatz 1, 10117 Berlin, Germany; 3 International Graduate Program Medical Neurosciences, Humboldt Graduate School, 10117 Berlin, Germany

**Keywords:** Migraine, Headache, Tau, NfL, Microglia, Biomarker, SIMOA, Cerebrospinal fluid

## Abstract

**Background:**

To date, migraine is diagnosed exclusively based on clinical criteria, but fluid biomarkers are desirable to gain insight into pathophysiological processes and inform clinical management. We investigated the state-dependent profile of fluid biomarkers for neuroaxonal damage and microglial activation as two potentially relevant aspects in human migraine pathophysiology.

**Methods:**

This exploratory study included serum and cerebrospinal fluid (CSF) samples of patients with migraine during the headache phase (ictally) (*n* = 23), between attacks (interictally) (*n* = 16), and age/sex-matched controls (*n* = 19). Total Tau (t-Tau) protein, glial fibrillary acidic protein (GFAP), ubiquitin carboxy-terminal hydrolase L1 (UCH-L1), and neurofilament light chain (NfL) were measured with the Neurology 4-plex kit on a Single Molecule Array SR-X Analyzer (Simoa® SR-X, Quanterix Corp., Lexington, MA). Markers of microglial activation, C-X3-C motif chemokine ligand 1 (CX3CL1) and soluble triggering receptor expressed on myeloid cells 2 (sTREM2), were assessed using an immunoassay.

**Results:**

Concentrations of CX3CL1 but not sTREM2 were significantly increased both ictally and interictally in CSF but not in serum in comparison to the control cohort (*p* = 0.039). ROC curve analysis provided an AUC of 0.699 (95% CI 0.563 to 0.813, *p* = 0.007). T-Tau in serum but not in CSF was significantly increased in samples from patients taken during the headache phase, but not interictally (effect size: η^2^ = 0.121, *p* = 0.038). ROC analysis of t-Tau protein in serum between ictal and interictal collected samples provided an AUC of 0.729 (95% CI 0.558 to 0.861, *p* = 0.006). The other determined biomarkers for axonal damage were not significantly different between the cohorts in either serum or CSF.

**Discussion:**

CX3CL1 in CSF is a novel potential fluid biomarker of migraine that is unrelated to the headache status. Serum t-Tau is linked to the headache phase but not interictal migraine. These data need to be confirmed in a larger hypothesis-driven prospective study.

**Supplementary Information:**

The online version contains supplementary material available at 10.1186/s10194-024-01757-8.

## Background

Migraine is among the most prevalent neurological disorders, with a one-year prevalence of about 15% [1, 2]. Currently, migraine is defined and diagnosed exclusively based on clinical criteria [3]. There are no clinical predictors or biomarkers to inform clinical management once the diagnosis of migraine is established, limiting personalized medicine strategies [4]. Fluid biomarkers are desirable to assist clinical decision-making in several instances, e.g., to monitor disease activity, potentially predict and stratify therapeutic approaches, and inform novel treatment targets [5]. Potential biomarkers of neuroinflammation are of interest, as migraine headaches, at least in part, reflect neurogenic inflammation along trigeminal afferents, including meningeal branches [6]. Overeem et al. recently described serum total Tau (t-Tau) as a potential novel biomarker possibly reflecting peripheral trigeminal neuroinflammation in migraine [7]. The state-dependent pattern of t-Tau release, however, remains to be determined. It is currently unknown if t-Tau is exclusively elevated in serum but not cerebrospinal fluid (CSF) during the headache phase and in neither compartment interictally. The latter would support the notion that its release is due to an activation of the trigeminovascular system and peripheral neuroinflammatory mechanisms. Biomarkers for neural destruction were not elevated in this or in previous migraine studies [7, 8]. Imaging studies using radioactive tracers and mouse models furthermore point towards a critical role of microglia in migraine pathophysiology [9, 10]. Microglia are essential mediators of neurogenic inflammation and promote the production of inflammatory and cytotoxic mediators [6]. A potential biomarker to mirror microglial activity is C-X3-C motif chemokine ligand 1 (CX3CL1), which is a chemokine released by neurons and glia. Its receptor, CX3CR1, is primarily expressed on microglia. CX3CL1 acts as a regulator of microglial activation within the central nervous system (CNS) [11]. Another biomarker for microglia activation is the triggering receptor expressed on myeloid cells 2 (TREM2). TREM2 is involved more generally in microglial activation in the brain [12, 13]. In CSF, sTREM2 is strongly correlated with CSF biomarkers of Tau pathology that track closely with the neurodegenerative processes in Alzheimer’s disease (AD) [14]. Neurodegenerative processes in migraine patients were discussed in view of white matter lesions and volumetric changes in white and grey matter on magnetic resonance imaging (MRI) [15].

In summary, there is an evolving set of candidate fluid biomarkers in migraine. Markers of microglial activation in CSF have not been tested to date in humans with migraine. This is not surprising since biomarker analyses in the CSF of migraine patients are rarely available as CSF is not routinely collected from migraine patients. The situation is further complicated by the necessity to determine the state-dependent profile of microglial activation, i.e., whether changes are found in the headache phase or interictally, in serum or CSF. For this purpose, we assembled a unique cohort of combined CSF and serum samples from migraine patients in their headache and interictal states, as well as age-matched controls. Based on the results of the study by Overeem et al. [7] data from these cohorts will furthermore be used to characterize the state-dependent profile of t-Tau release in migraine, which is critical to advance the understanding of its role in migraine pathophysiology.

## Methods

### Ethical approval and study registration

All procedures adhered to the *Helsinki Declaration* in its latest revision and were conducted in line with current guidelines for *good clinical practice*. This retrospective, single-center cohort study was based on the use of coded surplus material. The use of coded surplus material without additional informed consent was based on the local ethics research committee statement (ID: III UV 39/03 and BB 161/18, University Medicine Greifswald, Germany). All participants provided their consent that surplus biological samples may be used along with clinical data for research purposes.

### Study design and participant selection

This is a retrospective exploratory case-control study with blood and CSF samples of episodic migraineurs serving as cases and samples of patients without neurological diseases serving as matched controls. Matching was done for age (±5 years) and sex. There were several indications for CSF analysis. In patients with interictal migraine, suspected diagnoses were transient symptoms later diagnosed as aura (*n* = 6), migraine as a concomitant disease (*n* = 3), headache symptoms not previously classified as migraine (*n* = 7). Patients with ictal migraine received lumbar puncture because of headache characteristics different form previous presentations (*n* = 9), exclusion of infections in headache disorder not previously diagnosed as migraine (*n* = 13) and unclear aura symptoms (*n* = 1).

Control subjects had nonspecific symptoms and a neurological disorder was eventually excluded. Discharge reports were required not to include any evidence of a headache disorder (defined as a headache frequency of < 1/year and a negative medical history). Cerebrospinal fluid analysis in these cases was done to rule-out neuroinflammatory disorders that may have been undetected on MRI, although suspicion was generally low. But thorough work-up is warranted before diagnosing a patient with a functional disorder. The diagnosis of migraine was retrieved from discharge reports, re-reviewed, and confirmed by certified specialists from the University Headache Center according to international classification of headache disorders, 3rd revision (ICHD-3) criteria [3]. Importantly, evidence of recurrent attacks was required to confirm that patients suffer migraine as a headache disorder and not only a first-time migrainous headache. Insufficient clinical information following chart review or any doubt about the diagnosis led to the exclusion of the patient. Further exclusion criteria were the presence of any neurodegenerative, neuro-, or systemic inflammatory condition or any lesional defect in cerebral imaging. Concerning the detailed selection process of the patient samples see supplementary Fig. 1.

Minor parts of the analyses were previously used to assist in the interpretation of another data set; however, they were not the main focus of the current publication [7].

### Laboratory analyses

All samples were acquired from patients treated at the Department of Neurology, University Medicine of Greifswald (Greifswald, Germany). Lumbar CSF was collected according to local standards. CSF supernatant was extracted and aliquoted in 0.5 mL polypropylene tubes. Blood and CSF samples were stored at − 80 °C until measurement. All standard laboratory CSF analyses were performed as described previously [16].

We quantified t-Tau protein, glial fibrillary acidic protein (GFAP), ubiquitin carboxy-terminal hydrolase L1 (UCH-L1) and neurofilament light chain (NfL) with a Single Molecule Array (Simoa®, Neurology 4-plex A kit Lot No.: 503223) on the SR-X platform according to the manufacturer’s instructions (Quanterix, Billerica, MA, USA). Samples were analysed in duplicates following the manufacturer’s instructions and standard procedures at the department of neurology, University Medicine Greifswald. Serum samples were measured at a dilution of 1:4, CSF 1:100. The assays’ lower limit of quantification lies at 0.317 pg/mL (NfL), 0.933 pg/ml (GFAP), 9.6 pg/ml (UCH-L1), 0.114 pg/ml (t-Tau). The limit of detection (LOD) lies at 0.136 pg/mL (NfL), 0.276 pg/ml (GFAP), 4.03 pg/ml (UCH-L1), 0.0298 pg/ml (t-Tau). Three serum Tau samples were measured below the LOD. All other measurements were above these limits. Comparability was assured by including two plate controls (Lot Nr. 131,802) in all runs according to the manufacturer’s instructions. CX3CL1 was quantified with the QuantikineTM ELISA Human CX3CL1/Fractalkine Immunoassay (R&D Systems) according to manufacturer’s instructions. The limit of detection lies at 0.072 ng/mL (assay range: 0.2–10 ng/mL). s-TREM was quantified with the Human TREM2 SimpleStep ELISA Kit (abcam) according to the manufacturer’s instructions. The limit of detection lies at 10.5 pg/mL (assay range: 78.1 pg/ml - 5000 pg/ml). One patient sample presented values of CX3CL1 and sTREM2 below the LOD. Samples below the LOD were excluded from analysis.

### Data evaluation and statistics

Primary endpoint was the difference in absolute concentration in pg/ml of parameters microglial markers CX3CL1 and sTREM in serum and CSF samples between patients with migraine either collected during the acute migraine attack (ictal collection) or between attacks (interical collection) and healthy controls. Secondary parameters were markers for neuroaxonal destruction (NfL, GFAP, UCHL-1, t-Tau protein). Statistical analyses were carried out with SPSS (v28.0, IBM, Armonk, NY, USA) and GraphPad Software (version 7, La Jolla, CA). Normally distributed group data are presented as mean ± standard deviation; non-normally distributed data are presented as median and their interquartile range. Continuous data were analyzed for normal distribution using the Kolmogorov-Smirnov test. All biomarkers were non-normally distributed. Inferential comparisons within and between group means were carried out using the Mann-Whitney U test for pairwise comparisons and the Kruskal-Wallis test for more than two groups. Single parameters were post-hoc compared pairwise and corrected for multiple comparisons using the Bonferroni method. Frequencies are reported numerically. Classification properties of significant between-group differences of t-Tau protein (ictal vs. interictal state) and CX3CL1 (migraine vs. control) were assessed using receiver operating characteristic (ROC) analyses, the performance of which is reported by their area under the curve (AUC) and 95% confidence intervals (CI). ROC curve analysis was calculated with MedCalc® Statistical Software version 20.115 (MedCalc Software Ltd., Ostend, Belgium). *P*-values below 0.05 were considered significant.

### Data availability

The data that supports the findings of this study are available from the corresponding author, upon reasonable request.

## Results

### Demographics

We included blood and CSF samples of 39 different patients with episodic migraine (31 female, 35.4 ± 4.2 years of age), of which 16 were acquired interictal and 23 in acute migraine attacks. No patient was receiving preventive medication at the time of the lumbar puncture. 19 age-matched patients (12 female, 32.68 ± 10.29 years of age) were used as controls (Table 1). Age, sex, and albumin quotient did not differ between groups (all *p* > 0.05).

**Table 1:** Summary of biomarker levels of patients

	Controls (*n* = 19)	Migraine, interictal (*n* = 16)	Migraine, ictal (*n* = 23)	Significance (*p*-value)
**Age in years**	27 (25;41)	31.6 (26.5;41.7)	34.9 (22.4;42.7)	0.84
**Female**	*n* = 12 (63%)	*n* = 11 (69%)	*n* = 20 (87%)	0.21
**CSF/Serum QAlb**	5.3 (4.3;7.2)	4.85 (3.78;6)	4.5 (3.5;5.8)	0.583
**t-Tau CSF pg/ml**	42.96 (35.13;70.57)	39.54 (16.87;65.95)	51.17 (36.23;64.83)	0.42
**t-Tau Serum pg/ml**	0.43 (0.33; 0.54)^a^	0.3 (0.13;0.63)^a^	0.69 (0.39;1.07)^a^	0.038
**GFAP CSF pg/ml**	5340.77 (2905.59;6486.36)	4976.22 (3334.72;7233.63)	5050.26 (4373.31;5856.72)	0.999
**GFAP Serum pg/ml**	67.23 (57.84;111.04)	62.25 (38.53;99.93)	73.28 (52.63;81.11)	0.569
**UCHL1 CSF pg/ml**	752.17 (501.63;1170.98)	674.22 (603.45;781.81)	748.98 (602.21;845.07)	0.662
**UCHL1 Serum pg/ml**	13.89 (10.84;17.47)	16.51 (12.61;21.86)	14.82 (11.16;19.67)	0.573
**NfL CSF pg/ml**	324.59 (215.55;404.65)	297.895 (241.82;467.27)	281.4 (181.19;393.29)	0.644
**NfL Serum pg/ml**	4.42 (2.98;5.73)	3.88 (2.15;6.19)	3.599 (2.83;6.13)	0.834
**s-TREM CSF pg/ml**	25,947.34 (21,658.7;31,157.51)	26,591.31 (20,972.84;30,399.76)^a^	24,656.7 (16,693.72;30,423.62)	0.8
**s-TREM Serum pg/ml**	22,455.88 (17,568.14;32,755.89)	24,322.96 (15,644.24;29,010.35)^a^	19,409.59 (14,881.68;26,438.02)	0.55
**CX3CL1 CSF pg/ml**	0.15 (0.12;0.18)	0.18 (0.15;0.21)^a^	0.19 (0.16;0.24)	0.039
**CX3CL1 Serum pg/ml**	0.8 (0.72;0.92)	0.83 (0.65;1.07)^a^	0.87 (0.75;1.23)	0.445

Biomarkers levels of patients with migraine obtained during attacks and interictally, and age-matched controls. Continuous data are expressed as medians (first and third quartiles); nominal data are given as percentages, unless otherwise indicated. *P*-value was assessed with a Kruskal-Wallis test between all groups. ^a^ n-1 (one sample each was measured below the LOD)

### Total tau protein in serum but not in CSF is elevated exclusively in the headache phase

**Fig. 1:**
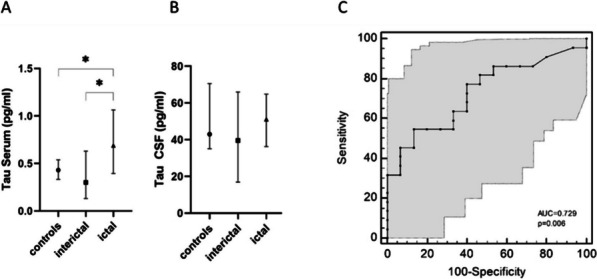
Box plots of the temporospatial profile of t-Tau. The median and IQR of t-Tau protein concentration in pg/ml in serum and CSF are presented. There is a significant difference in t-Tau protein concentration in serum between the interictal collected sample and the ictal collected sample of migraine patients (*p < 0.05). ROC curve analysis between the ictal and interictal collected migraine samples provided an AUC of 0.729 [AUC = 0.729 (95% CI 0.558 to 0.861, *p* = 0.006)]

As seen in Fig. 1, there was a significant global effect comparing all 3 groups (effect size: η^2^ = 0.121, *p* = 0.038). Post-hoc tests yielded that this was due to a significant difference between samples obtained in the headache phase and interictally (*p* = 0.037) (Fig. 1A). In contrast, this effect cannot be detected in CSF, where irrespective of the migraine state concentrations correspond to the level of the control cohort (*p* = 0.42, Fig. 1B). ROC analysis between migraine samples obtained in the headache and interictal phase yielded an AUC of 0.729 [AUC = 0.729 (95% CI 0.558 to 0.861, *p* = 0.006)] (Fig. 1C).

### Biomarkers for axonal damage are not elevated in migraine patients

**Fig. 2:**
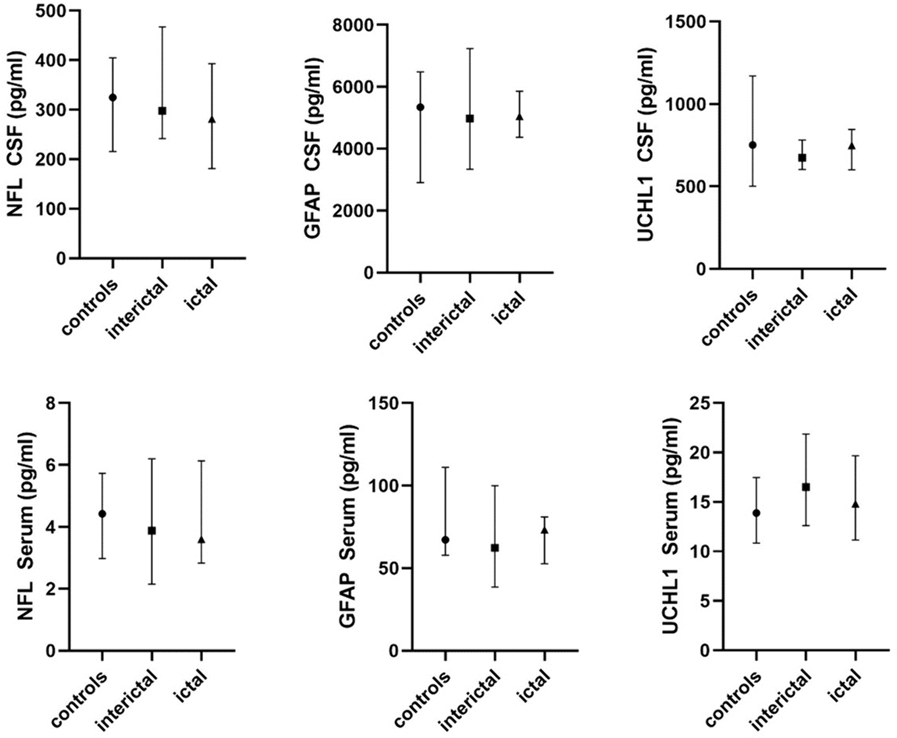
Box plots of markers of axonal damage. The median and IQR of NfL, GFAP, and UCHL1 concentrations in pg/ml in CSF (upper row) and serum (lower row) of the 3 different cohorts are presented. There is no significant difference in any protein concentration, neither in serum nor in CSF, between the interictal, the ictal-collected sample of migraine patients, or the control cohort of non-migraine patients (for all *p* > 0.05)

Other biomarkers for axonal damage such as GFAP, UCHL-1 and NfL concentrations were not significantly different in either serum (Fig. 2, upper row) or CSF (Fig. 2, lower row) between the 3 different cohorts (serum GFAP *p* = 0.341; CSF GFAP *p* = 0.993; serum NfL *p* = 0.579; NfL CSF *p* = 0.98; serum UCHL-1: 0.431; UCHL-1 CSF: 0.579).

### Microglial activation marker CX3CL1, but not sTREM2, is elevated in CSF only in migraine patients

**Fig. 3:**
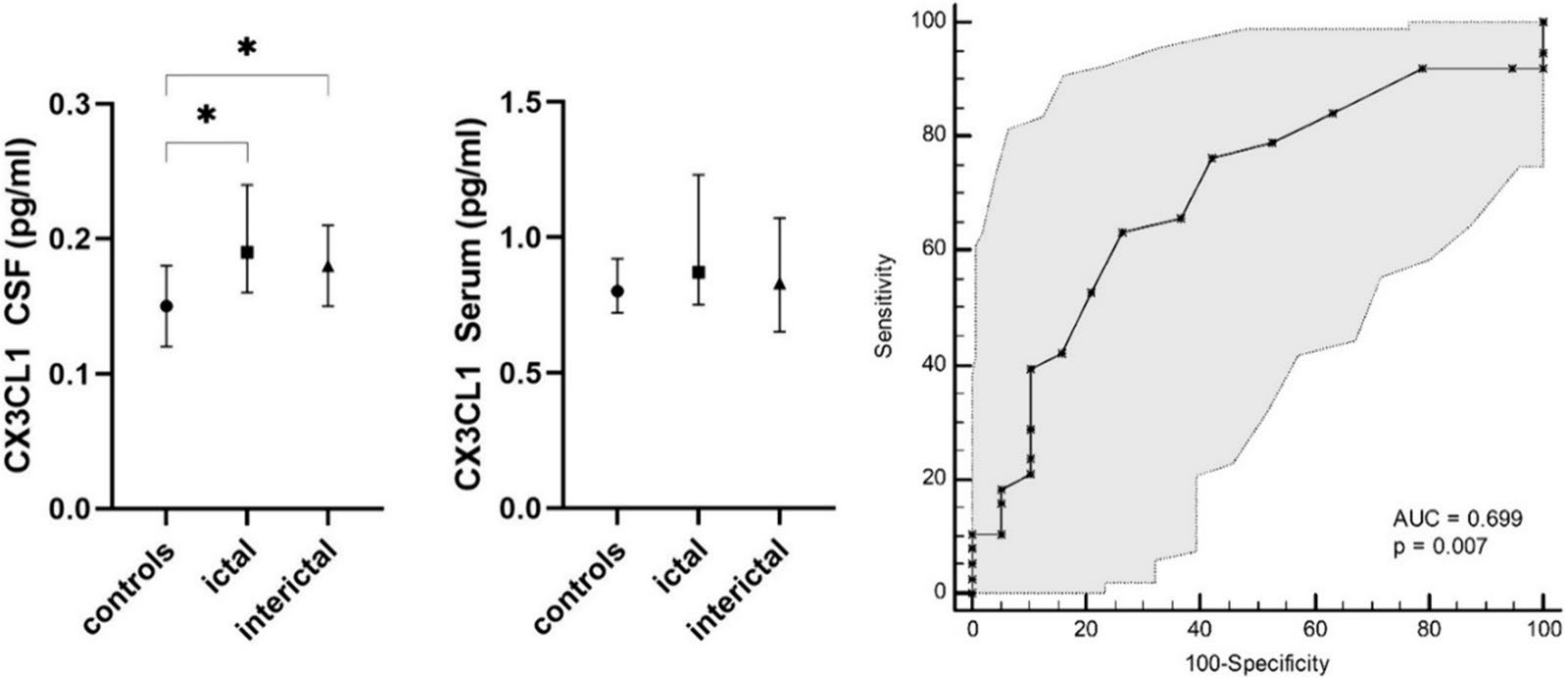
Box plots of the temporospatial profile of CX3CL1. The median and IQR of CX3CL1 concentration in pg/ml in serum and CSF of the 3 different cohorts are presented. A significant difference was found in the amount of CX3CL1 in the cerebrospinal fluid (CSF) between the sample of migraine patients who had an ictal headache and the control group and of migraine patients who did not have an ictal headache. Right: ROC curve analysis of CX3CL1 between migraine patients (ictally and interictally collected samples) and healthy controls, AUC of 0.699 (95% CI 0,563 to 0,813, *p* = 0.007)

**Fig. 4:**
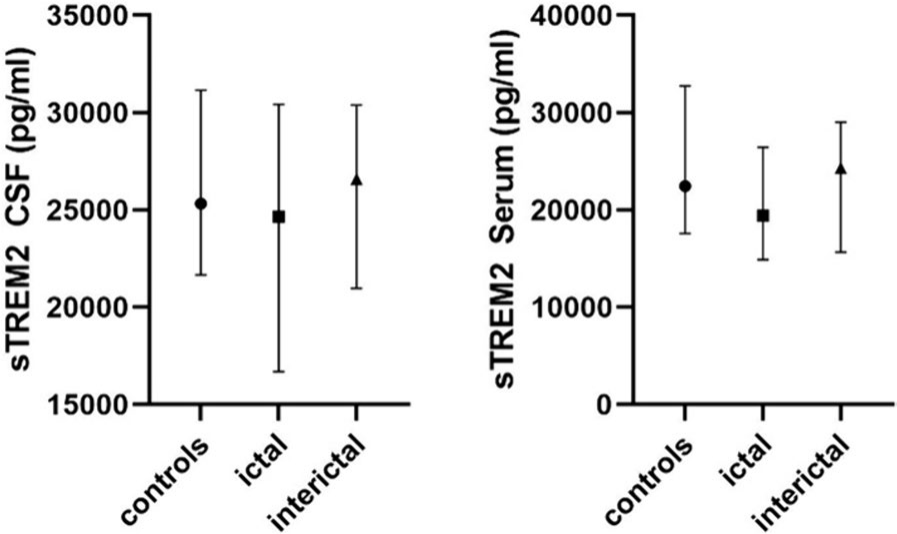
Box plots of the temporospatial profile of sTREM2. The median and IQR of sTREM2 concentration in pg/ml in serum and CSF of the 3 different cohorts are presented. STREM2 levels did neither differ between controls and patients with migraine nor between different stages of migraine

Concentrations of CX3CL1 were significantly increased both in the headache phase and interictally in CSF but not in serum compared with the non-migraine cohort (*p* = 0.014) (Fig. 3). The AUC to differentiate between samples of patients with migraine, both in the headache phase and interictally, and non-migraine samples was 0.699 (95% CI 0.563 to 0.813, *p* = 0.007) (Fig. 3). There was no significant difference between the concentration of CX3CL1 in the headache phase and interictally (*p* = 0.442). There was no significant difference in the concentrations of sTREM2 as an additional biomarker for microglial activation, neither in serum nor in CSF, between the 3 cohorts (Fig. 4).

## Discussion

This is the first study to demonstrate that microglial activation can be detected in patients with migraines, irrespective of their headache status. CX3CL1 in CSF thus provides a novel biomarker to study migraine pathophysiology in humans. We were furthermore able to clarify the state-dependent effects of serum t-Tau in the migraine cycle and found that its concentrations were elevated in the headache phase but not interictally, rendering it a potential biomarker of migraine attacks. T-tau elevations were not accompanied by an increase in other biomarkers for neurodegeneration, which argues against significant neuroaxonal deterioration in migraine patients. In line, the absence of an increase in GFAP in migraine patients, as well as the lack of an increase in NFL speaks against astrocytic or neuroaxonal damage in the CNS in migraine patients.

In contrast, CX3CL1 and t-Tau pose potential fluid biomarkers to study the pathophysiology of migraine and possibly inform clinical management.

### Serum t-tau as a potential biomarker for disease activity in migraine patients

Tau protein in peripheral blood samples has been tested as a marker of neuroaxonal damage in a variety of diseases [17]. Only one study investigated the significance of t-Tau in the serum of migraine patients in the interictal phase of the disease. Overeem et al. found increased concentrations of t-Tau in migraine patients compared with healthy controls [7]. Based on our data, we demonstrated that this difference is driven primarily by elevated concentrations in the headache phase of the disease. However, according to the paper by Overeem et al., there is no dependence of t-Tau release on the presence of aura symptoms [7]. The authors conclude that it is not cortical activity but neuronal pain-associated activity, e.g. in the trigeminal ganglion, that could be responsible for t-Tau release. Our finding of serum t-Tau elevation in the ictal phase of the disease supports this hypothesis. In conclusion, serum t-Tau could be a potential biomarker for disease activity, as it is already used in peripheral neuroinflammatory disorders such as Guillain-Barré syndrome [18]. Further studies are needed to evaluate the relationship between attack frequency, distance to migraine attacks, and dependence on specific migraine therapeutics and concentration of t-Tau protein in serum.

We demonstrated that t-Tau concentration is detectable only in serum and not in CSF. This offers room for pathophysiologically relevant speculation in the context of migraine attacks. It was speculated that t-Tau elevation in migraine patients represents functional changes in the trigeminal nervous system [7], but without further elucidation of a specific mechanism. Regardless of why t-Tau protein elevation occurs in migraine patients, the lack of difference in concentrations of other biomarkers of neuroaxonal degeneration such as NfL, GFAP and UCHL1 needs to be emphasized and refutes major neurodegenerative processes in migraine.

### CX3CL1 in CSF as a biomarker for microglial activity in migraine patients

CX3CL1 in CSF but not in serum was significantly higher in migraine patients regardless of sample collection in the headache phase or interictally versus healthy controls. Even though there are some investigations in mouse models suggesting microglial activation in migraine, evidence in human subjects is lacking [19]. Most murine studies investigated purinergic receptors (e.g., P2X/P2Y), which is plausible given the metabolic aspects of migraine pathophysiology, including neuronal energy deficiency [20]. The involvement of the fraktalkine pathway was only recently suggested in another mouse model of migraine and was also associated with impaired neuronal signalling in thalamo-cortical networks [21]. We found that CX3CL1, the ligand to the fractalkine receptor, is increased in humans irrespective of the headache status. Based on the current evidence, it can only be speculated that increased microglial activity could represent a pro-neuroinflammatory trait, with implications for neuronal transmission in regions with dense microglial concentration such as the thalamus [21]. Additionally, microglia are thought to play an important role in the modulation of migraine, and this process may be responsible for the progression toward chronification [19]. Also, microglia activation has been associated with cortical spreading depolarization [22], a potential cause of microgliosis in migraine patients. Supporting this notion, patients undergoing CSF examination in the interictal phase of migraine in this study mostly presented with transient neurological symptoms and white matter abnormalities on MRI, which were eventually attributed to migraine with aura. It remains to be clarified whether CX3CL1 concentrations differ between patients with migraines with and without aura. Finally, neuron-microglia interactions play a role in pain generation associated with migraine [23, 24]. In our study, we detected an increase in CX3CL1 in comparison with healthy controls solely in the CSF, which is indicative of a central origin of CX3CL1.

Interestingly, we could not detect a difference in the levels of another microglial marker, sTREM2, between the groups studied. To date, no studies exist on the significance of sTREM2 in the context of migraine. Given that there are no indications of neurodegeneration in migraine, neither in neuroimaging nor in biomarker studies, a lack of elevations of sTREM2, which is associated with microglial activation in Alzheimer’s and Parkinson’s disease, is plausible [25]. It must be noted that micoglial activation is indeed not a simple binary state but rather a fine-tuned homeostatic process that may differ between the site of micoglial activation and its triggering receptor [26].

### Limitations

A main limitation of this study is the retrospective design, so that no more detailed information on further migraine characteristics could be collected, such as duration of the disease, or acute or preventive medication. On the other hand, CSF samples were specifically acquired in order to rule out other neurological disorders. Performing a lumbar puncture is nowadays rather unusual in migraine patients making it almost impossible to perform prospective CSF studies in this population. For the same reason, a higher number of cases is very difficult to achieve, even if this would certainly be desirable for a biomarker analysis, particularly with regard to the statistical significance between the individual groups. Body mass index (BMI) may be a covariate for at least NFL and GFAP; no values were available for this because of the retrospective design. For other covariates, such as age and gender, we controlled by choosing an age- and gender-matched control group.

Another limiting factor is the sometimes very long storage time of the samples before the above-mentioned biomarkers have been measured. On the other hand, it has been described that the measurement of t-Tau is reliable even after years of storage [27].

To assess the levels of the above biomarkers as diagnostic, other control groups would need to be studied. Since this study was exploratory in nature, larger studies with other patient cohorts would be desirable, specifically for t-Tau protein in serum.

To assess disease activity by a biomarker, longitudinal sample collections from the same patients would be desirable. This is also a goal of future studies.

## Conclusion

CX3CL1 in CSF and serum t-Tau are novel potential fluid biomarkers in migraine, which may advance the investigation of migraine pathophysiology and inform clinical management. These data need to be confirmed on larger hypothesis-driven prospective study cohorts.

### Supplementary Information

**Additional file 1: Suppl. Fig.** **1** Flow chart of patient sample selection.

## Data Availability

The datasets generated and/or analysed in the current study are not publicly available due to data protection regulations that impede unconditional distribution. The data that supports the findings of this study are, however, available from the corresponding author upon reasonable request.
