# Supplementary figures and images for: Increased CX3CL1 in cerebrospinal fluid and ictal serum t-tau elevations in migraine: results from a cross-sectional exploratory case-control study
Source: J Headache Pain. 2024 Apr 2;25(1):46. doi: 10.1186/s10194-024-01757-8 (PMC10985871; doi:10.1186/s10194-024-01757-8)

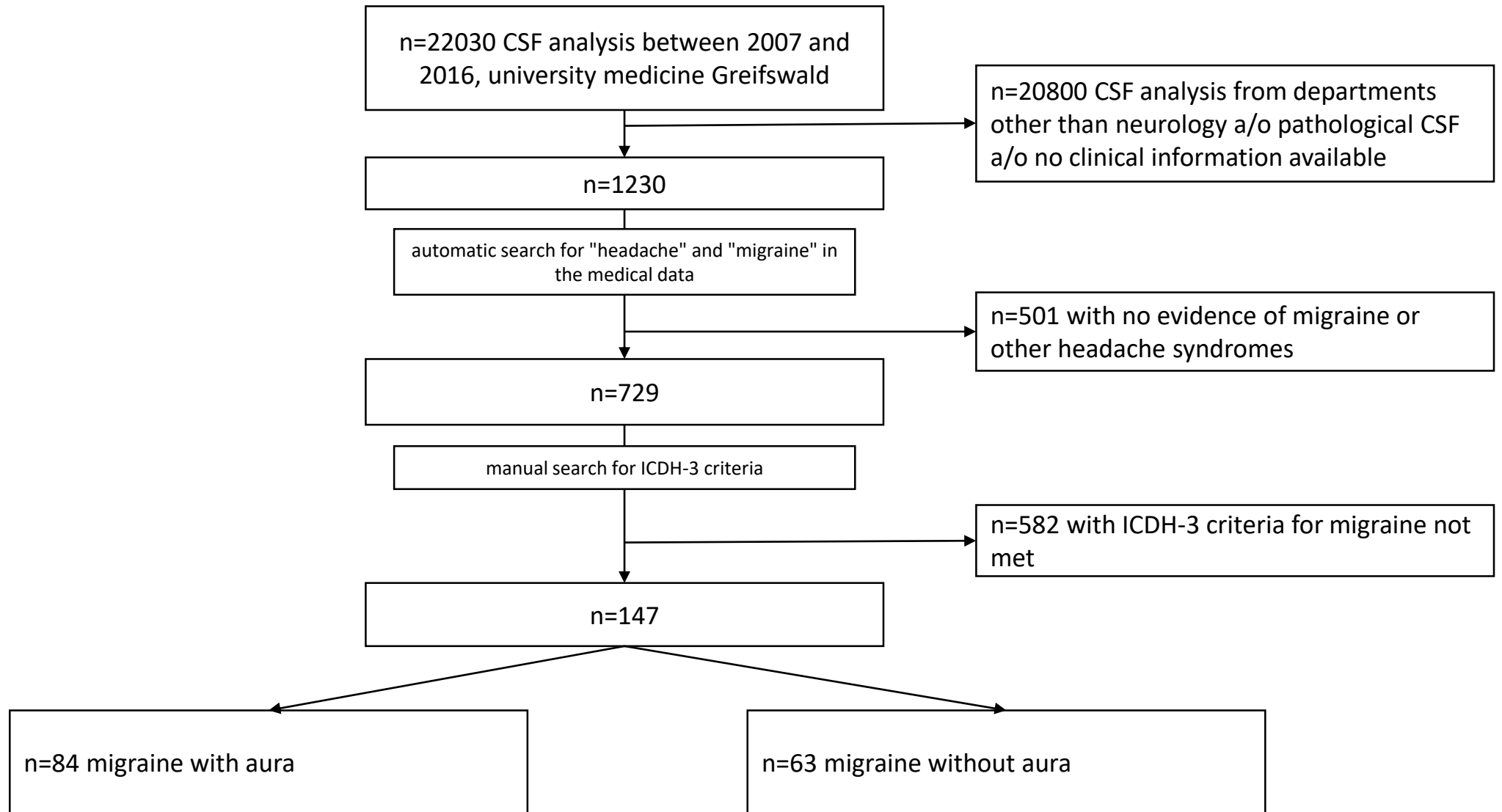

Supplement: Supplementary file 1 — Additional file 1: Suppl. Fig. 1 Flow chart of patient sample selection. [file 10194_2024_1757_MOESM1_ESM.pdf]
